# Reduced phonemic fluency in progressive supranuclear palsy is due to dysfunction of dominant BA6

**DOI:** 10.3389/fnagi.2022.969875

**Published:** 2022-09-08

**Authors:** Valeria Isella, Daniele Licciardo, Francesca Ferri, Cinzia Crivellaro, Sabrina Morzenti, Ildebrando Appollonio, Carlo Ferrarese

**Affiliations:** ^1^Department of Neurology, School of Medicine, University of Milano - Bicocca, Monza, Italy; ^2^Milan Center for Neurosciences, Milan, Italy; ^3^Neurology Unit, San Gerardo Hospital, Monza, Italy; ^4^Nuclear Medicine Unit, San Gerardo Hospital, Monza, Italy; ^5^Medical Physics, San Gerardo Hospital, Monza, Italy

**Keywords:** supplementary motor area, frontal aslant tract, progressive supranuclear palsy, fluency, FDG-PET

## Abstract

**Background:**

Reduced phonemic fluency is extremely frequent in progressive supranuclear palsy (PSP), but its neural correlate is yet to be defined.

**Objective:**

We explored the hypothesis that poor fluency in PSP might be due to neurodegeneration within a dominant frontal circuit known to be involved in speech fluency, including the opercular area, the superior frontal cortex (BA6), and the frontal aslant tract connecting these two regions.

**Methods:**

We correlated performance on a letter fluency task (F, A, and S, 60 s for each letter) with brain metabolism as measured with Fluoro-deoxy-glucose Positron Emission Tomography, using Statistical Parametric Mapping, in 31 patients with PSP.

**Results:**

Reduced letter fluency was associated with significant hypometabolism at the level of left BA6.

**Conclusion:**

Our finding is the first evidence that in PSP, as in other neurogical disorders, poor self-initiated, effortful verbal retrieval appears to be linked to dysfunction of the dominant opercular-aslant-BA6 circuit.

## Introduction

The ability to retrieve words upon a phonemic cue is a cognitively complex task that relies mostly on executive functions (Lezak et al., 2012; Shao et al., 2014) and depends predominantly on the integrity of the frontal lobes. Studies and meta-analyses carried out in healthy subjects with structural and functional Magnetic Resonance Imaging (MRI) or neurophysiological techniques have in fact highlighted the prominent involvement of the left inferior/middle frontal gyrus and insula and of bilateral anterior cingulate in phonemic fluency (PF) tasks (Devlin et al., 2003; Shibahara, 2004; Tremblay et al., 2004; Iyer et al., 2005; Costafreda et al., 2006; Heim et al., 2008; Meinzer et al., 2009, 2012; Cattaneo et al., 2011; Katzev et al., 2013; Wagner et al., 2014; Yuan and Raz, 2014; Smirni et al., 2017; Ghanavati et al., 2019; Vonk et al., 2019; Oswald et al., 2022). Neuroimaging studies in patients with degenerative cognitive or movement disorders such as Alzheimer’s disease (Melrose et al., 2009; Clark et al., 2014; Rodríguez-Aranda et al., 2016; Jones et al., 2019), corticobasal syndrome (Parmera et al., 2021), and frontotemporal dementia and primary progressive aphasias (Robinson, 2006; Crescentini et al., 2008; Laisney et al., 2009; Cook et al., 2014; Robinson et al., 2015; Suppa et al., 2020; Riello et al., 2022) have generally confirmed that impairment of PF is associated with left inferior frontal dysfunction, but have also indicated the involvement of other left cortical and subcortical structures like the temporal lobe (Laisney et al., 2009; Libon et al., 2009; Cook et al., 2014; Rodríguez-Aranda et al., 2016; Parmera et al., 2021; Riello et al., 2022), the superior medial frontal cortex (Robinson, 2006; Robinson et al., 2015) and the basal ganglia (Robinson, 2006; Crescentini et al., 2008; Melrose et al., 2009; Clark et al., 2014), and of right, mainly frontal, regions (Libon et al., 2009; Melrose et al., 2009; Clark et al., 2014; Riello et al., 2022).

Among all these neural loci, a complex of left frontal areas has consistently shown a relationship with PF in both healthy individuals and neurological, not only degenerative, patients (Robinson et al., 2012; Catani et al., 2013; Chapados and Petrides, 2013; Kinoshita et al., 2015; Rodríguez-Aranda et al., 2016; Li et al., 2017; Miró-Padilla et al., 2017; Blecher et al., 2019; Dick et al., 2019; Jones et al., 2019; Vonk et al., 2019; Keser et al., 2020; Suppa et al., 2020; La Corte et al., 2021; Parmera et al., 2021; Pinson et al., 2022), which includes the inferior gyrus—pars opercularis—and insula, the superior gyrus corresponding to BA6 and comprising the supplementary motor area (SMA), and the white matter tract that connects these two inferior and superior frontal regions, i.e., the frontal aslant tract (FAT) (Catani et al., 2012).

Reduced PF is one of the cognitive hallmarks of progressive supranuclear palsy (PSP). It is present in up to 85% of PSP patients irrespective of the specific phenotype, often from a very early disease stage (Kertesz and McMonagle, 2010; Burrell et al., 2014; Gerstenecker, 2017; Pellicano et al., 2017; Peterson et al., 2021), and appears to be also quite specific: most of the studies that compared PF performance in patients with PSP, Alzheimer’s disease or extrapyramidal disorders like Parkinson’s disease, corticobasal syndrome and multiple system atrophy have shown more frequent and severe impairment in PSP (Cordato et al., 2006; Rittman et al., 2013; Lee et al., 2016; Gerstenecker, 2017; Pellicano et al., 2017; Peterson et al., 2021).

Since the frontal lobes are one of the main loci of neurodegeneration in PSP (Stamelou et al., 2021), it is not surprising that a primarily frontal ability like PF is often impaired in this disorder. Nevertheless, not many studies have up to now investigated the specific neural correlate of Letter fluency in individuals with PSP. The current study was aimed at investigating the hypothesis that neurodegeneration within the left frontal opercular-aslant-BA6 complex underlying speech fluency could account for reduced PF in these patients, using 18-Fluoro-Deoxy-Glucose Positron Emission Tomography (FDG-PET). A hint that this might be the case has come from a study that found a relationship between poor Letter fluency and abundant tau deposits in the superior frontal cortex in the brains of 11 subjects with PSP-RS (Schofield et al., 2012). Another study that investigated the neural substrate of poor fluency in this disease found a relation between PF and midbrain atrophy on MRI, but did not explore the frontal lobes (Luca et al., 2021). In our PSP sample we expected to find reduced FDG uptake in regions typically affected by degenerative processes in this disorder (the frontal cortex, basal ganglia, and midbrain), and lower left dorsal inferior and superior frontal metabolism for lower fluency scores.

## Methods

### Participants

Participants were recruited from the memory clinic and movement disorders clinic of San Gerardo Hospital, Monza. Inclusion criteria were a diagnosis of PSP according to standardized criteria (Höglinger et al., 2017) and Italian as native language. We excluded patients with a primary progressive aphasia presentation, in order to avoid the confounding effect of language impairment on the fluency task. Other exclusion criteria were moderate-to-severe vascular burden on brain imaging, and history of other neurological disorders, major psychiatric diseases, brain injury, substance abuse, developmental intellectual, or cognitive disorders.

All participants signed an informed consent before taking part in the study. The study was conducted according to the guidelines of the World Medical Association Declaration of Helsinki, and approved by our institution’s ethics committee, Comitato Etico Brianza.

### Neuropsychological assessment

Patients underwent a general neuropsychological battery that included the MiniMental State Examination [MMSE (Folstein et al., 1975; Measso et al., 1993)], and tests of selective attention [Attentional Matrices (Spinnler and Tognoni, 1987)], short-term memory [Digit span (Monaco et al., 2013)], verbal long term memory [Rey Auditory Verbal Learning Test, RAVLT (Carlesimo et al., 1996)], language production [Category fluency (Zarino et al., 2014)], visuo-constuctional abilities [copy of Rey-Osterrieth Complex Figure, ROCF (Caffarra et al., 2002)], limb apraxia [De Renzi test of Ideomotor Apraxia, IMA (De Renzi et al., 1980)], and executive functions [Frontal Assessment Battery (Appollonio et al., 2005), Raven Colored Progressive Matrices (Basso et al., 1987)]. Mood and behavior were assessed with the Neuropsychiatric Inventory (Cummings, 1997).

The Letter fluency test (Costa et al., 2014) was administered as part of the general neuropsychological battery. In this test Patients are instructed to produce words starting with letters F, A, and S, avoiding proper nouns like names of people or places, and numbers, in three 60-s trials. Repetitions, perseverations and series of the same word with a different suffix are considered errors. The test score is the sum of all correct words produced for all three letters.

### Acquisition, processing and analysis of metabolic imaging

18-Fluoro-Deoxy-Glucose Positron Emission Tomography scans were performed on a General Electric Discovery LS PET/CT scanner on average 2.9 ± 3.6 months within cognitive testing. After acquisition of CT images for attenuation correction, PET images were acquired for 15 min, with a thickness of 3.27 mm and a matrix of 128 × 128 pixels, and reconstructed following an ordered subset expectation maximization algorithm.

Processing of images was performed with Statistical Parametric Mapping (SPM) 8 (Wellcome Department of Imaging Neuroscience, London, United Kingdom)^1^ running on MATLAB R2015a (MathWorks Inc., Sherborn, MA, United States). Images were reoriented along the anterior–posterior commissure, spatially normalized to the Montreal Neurological Institute (MNI) reference space using an FDG-PET dementia-specific template (Della Rosa et al., 2014), and smoothed with an isotropic 3D Gaussian kernel of 16 mm FWHM.

Progressive supranuclear palsy patients’ scans were compared with scans from 30 neurologically healthy controls (disease-free oncologic patients undergoing PET for disease staging, 14 women, with a mean age of 71.3 years ± 7.7 and a mean MMSE score of 28.9 ± 1.2), using 2-sample *t*-test, including age and sex as covariates.

Association between brain metabolism and score on the letter fluency task and on copy of ROCF was assessed only within the group of PSP patients using “Multiple regression” in SPM, including age and sex as covariates of no interest. In order to control for the risk of a non-causal relationship, linked to the fact that cognition and brain metabolism are both declining in neurodegenerative patients, we also correlated FDG uptake with a neuropsychological task with no presumed relationship with the dominant frontal lobe, i.e., copy of ROCF.

Significance threshold was set at *p* < 0.05 Family Wise Error (FWE)-corrected or *p* < 0.001 uncorrected, and minimum cluster size was set at 100 voxels. Anatomical labeling of significant clusters was performed with Talairach and Automatic Labeling atlases integrated in SPM8 toolbox WFU_PickAtlas.

## Results

### Clinical and metabolic imaging characteristics of the progressive supranuclear palsy sample

The study sample was composed by 31 PSP patients, whose socio-demographic and neurological features are shown in Table 1. The most prevalent phenotype was PSP with predominant frontal presentation (n. 12/31, 38.7%), followed by Richardson’s Syndrome (n. 10, 32.3%), PSP with predominant parkinsonism (n. 8, 25.8%) and PSP with predominant postural instability (n. 1, 3.2%). Nearly all patients showed cognitive/behavioral frontal symptoms and/or signs of parkinsonism, and approximately 70% showed postural instability and/or ocular motor dysfunction, while language deficits and apraxia were less frequent. Biomarkers for amyloid status were available in a minority of cases [n. 9, 29.0%, cerebrospinal fluid (CSF) in eight, PET with amyloid-tracer in one], and were all indicative of non-amyloid pathophysiology.

**TABLE 1:** Sociodemographic and clinical characteristics of the progressive supranuclear palsy (PSP) sample.

Sex (men-women)	n. 16-15
Age	73.5 ± 7.5
Education (years)	8.2 ± 3.6
Disease duration (years)	2.8 ± 1.3
**Prevalence of neurological features:**	
Ocular motor dysfunction	n. 21, 67.7%
Postural instability	n. 22, 71.0%
Parkinsonism	n. 28, 90.3%
Frontal symptoms	n. 28, 90.3%
Language deficits	n. 19, 61.3%
Orobuccal/limb apraxia	n. 13, 42.0%

Mean ± SD, unless otherwise stated.

The group’s general neuropsychological profile and performance on the Letter fluency test are reported in Table 2. MMSE scores indicated that most patients had mild global cognitive impairment; the largest number of abnormal scores, based on published norms, was observed for copy of ROCF and executive and attentional tasks. More than 70% of patients showed an abnormal performance on Letter fluency.

**TABLE 2:** Neuropsychological profile of the PSP sample and performance of the Letter fluency test.

	Mean ± SD	Cases with abnormal scores*
MMSE	21.9 ± 4.5	n. 17, 54.8%
Letter fluency (F, A, S)	10.1 ± 6.1	n. 22, 71.0%
Attentional Matrices	28.0 ± 10.4	n. 18, 58.1%
Digit span	4.1 ± 1.1	n. 13, 41.9%
RAVLT immediate recall	21.9 ± 10.6	n. 18, 58.1%
RAVLT delayed recall	4.0 ± 3.3	n. 15, 48.4%
Category fluency	19.6 ± 7.2	n. 15, 48.4%
Frontal Assessment Battery	9.7 ± 3.6	n. 24, 77.4%
Raven CPM	18.1 ± 6.5	n. 8, 25.8%
Copy of ROCF	17.6 ± 9.4	n. 25, 80.6%
IMA Test (left + right arm)	114.9 ± 24.5	n. 10, 32.3%
NPI (total score)	12.7 ± 14.5	n. 26, 83.9%**

CPM, colored progressive matrices; IMA, ideomotor apraxia; MMSE, MiniMental State Examination; NPI, neuropsychiatric inventory; RAVLT, Rey Auditory Verbal Learning Test; ROCF, Rey-Osterrieth Complex Figure. *Based on published norms. **Cases with a score ≥1.

**FIGURE 1:**
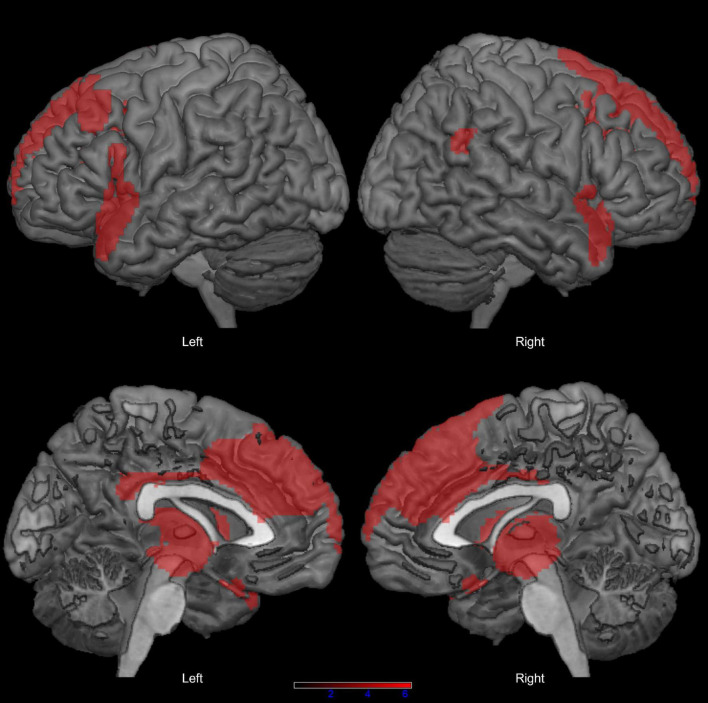
Distribution of hypometabolism in patients with progressive supranuclear palsy (PSP) compared with healthy controls (*p* < 0.05 family-wise-error corrected, minimum cluster size: 100 voxels).

We found no significant difference in sex distribution (x_2_ = 0.015, *p* = 1.000) and age (*t* = 1.547, *p* = 0.127) between the PSP group and the neuroimaging control group. In comparison with controls, patients with PSP showed extensive bilateral hypometabolism in the dorsolateral and mesial prefrontal cortex, inferior frontal and fronto-temporal regions, basal ganglia, and midbrain, at *p* < 0.05 FWE-corrected (Figure 1; see also Supplementary Table 1 for peak coordinates, clusters extent, and *t* and *p* values resulting from SPM analysis).

### Correlation between phonemic fluency and brain metabolism

**FIGURE 2:**
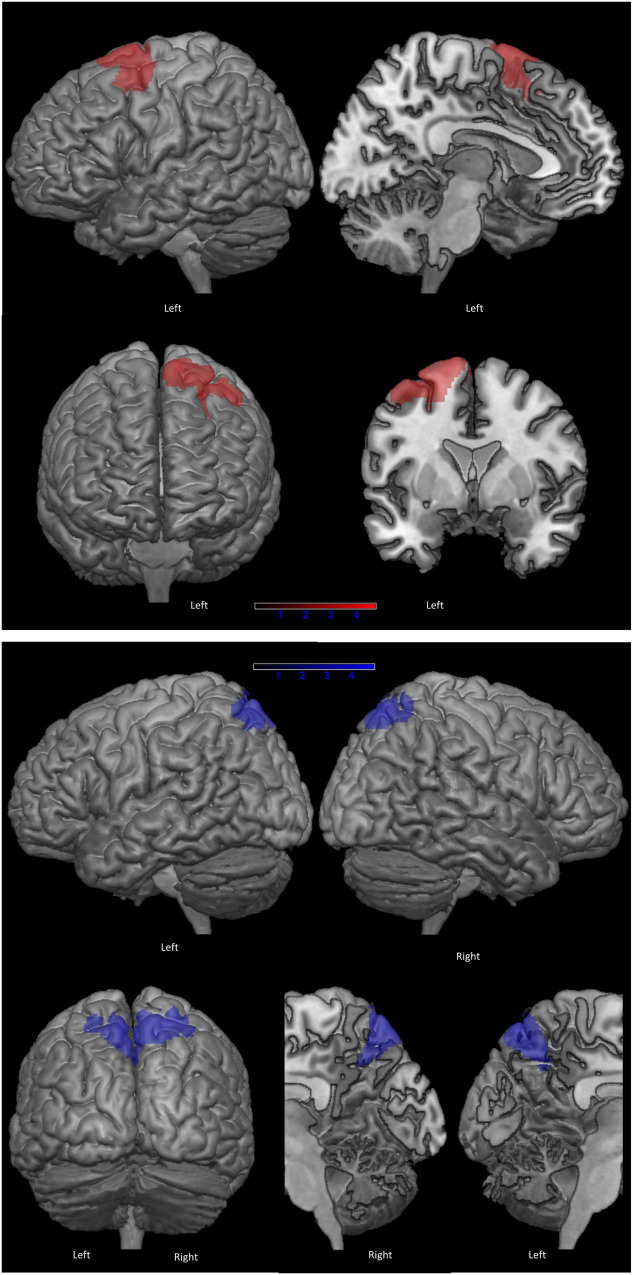
Clusters of hypometabolism correlated with phonemic fluency (in red) and with copy of Rey-Osterrieth Complex Figure (ROCF), as control task (in blue) (*p* < 0.001 uncorrected, minimum cluster size: 100 voxels).

Figure 2 shows the clusters of lower brain metabolism associated with lower scores on PF (in red) and copy of ROCF (in blue), both significant at *p* < 0.001 uncorrected. The neurometabolic correlate of PF performance was a 1,544 voxel cluster lateralized to the left hemisphere, and involving mainly the SMA (BA6) and the lateral surface of the superior-middle frontal gyri. The neurometabolic correlate of ROCF performance was a 1,910 voxel cluster encompassing the superior parietal cortex bilaterally. Supplementary Table 2 reports details for both SPM analyses.

## Discussion

Impairment of PF is extremely common and a very early sign of cognitive dysfunction in PSP. Although more frequent in Richardson’s Syndrome (Burrell et al., 2014; Peterson et al., 2021), and exacerbated by speech deficits in PSP-related progressive aphasia, it is present in most PSP phenotypes (Kertesz and McMonagle, 2010; Burrell et al., 2014; Gerstenecker, 2017; Peterson et al., 2021). Indeed the Movement Disorders Society criteria have cataloged poor letter fluency among the core frontal cognitive features of the disease (Höglinger et al., 2017). Neuroimaging studies in patients with focal brain damage or neurodegenerative disorders and in young and old healthy individuals have consistently shown a relationship between poor PF and left frontal dysfunction, in particular within two sets of regions: (i) the inferior gyrus (BAs) (Kertesz and McMonagle, 2010; Gerstenecker, 2017; Peterson et al., 2021) and insula, and (ii) the middle gyrus (BA) (Burrell et al., 2014), lateral and mesial superior gyrus including the SMA (BA6), and anterior cingulate (BA9) (Chapados and Petrides, 2013; Miró-Padilla et al., 2017; Vonk et al., 2019; Pinson et al., 2022). Importantly, these opercularis and superior frontal regions are interconnected through the FAT (Catani et al., 2012), whose damage has been shown to account for decreased verbal fluency in various neurological disorders (Catani et al., 2013; Chapados and Petrides, 2013; Kinoshita et al., 2015; Li et al., 2017; Blecher et al., 2019; Dick et al., 2019; Keser et al., 2020; La Corte et al., 2021; Pinson et al., 2022).

Results of the current study suggest that dysfunction within this left opercular-aslant-BA6 complex accounts for impairment of PF also in PSP. In agreement with the autopsy study mentioned in the Introduction, which showed a relationship between a higher degree of tau deposits in the left superior frontal cortex and worse fluency (Schofield et al., 2012), our correlation analysis between Letter fluency and brain metabolism in 31 patients with PSP identified a very definite locus of dysfunction centered around left BA6, and including both the SMA and the lateral superior frontal cortex. The same analysis on copy of ROCF yielded a different correlate [placed in the superior parietal cortex, known to be involved in visuoconstructional abilities (Raimo et al., 2021)], ruling out the possibility that the association between PF and BA6 was coincidental, i.e., due to a parallel, but causally unrelated, progression of cognitive impairment and hypometabolism.

The dominant mesial and lateral superior frontal cortex, and the SMA in particular, is thought to modulate the activity of the pars opercularis of the inferior frontal gyrus, via the FAT, during speech production tasks involving self-initiation, effortful retrieval, competition resolution, and monitoring (Alario et al., 2006; Catani et al., 2013; Kinoshita et al., 2015; Dick et al., 2019; Ardila, 2020). All functions engaged in Letter fluency, which relies on response initiation and inhibition, development and implementation of a lexical retrieval strategy, shifting, and output supervision (Lezak et al., 2012; Shao et al., 2014).

As the frontal lobes are the main cortical locus of neurodegeneration in PSP (Williams et al., 2007; Dickson et al., 2010), the dysfunction of the opercular-aslant-BA6 complex is not surprising, and probably has a multifactorial origin: tau deposits, neuronal and axonal loss, and also glial pathology are all typical findings in PSP brains (Zhukareva et al., 2006; Williams et al., 2007; Dickson et al., 2010). With the present study we have demonstrated the presence of synaptic dysfunction at the level of the superior frontal cortex related to poor PF. In the past, structural imaging studies showed diffuse white matter abnormalities in PSP (Agosta et al., 2010; Sajjadi et al., 2013), but the direct involvement of the FAT is yet to be investigated.

Future research will also have to overcome some of the limitations of our study. First of all, our sample was large enough for detecting an extensive area of significantly reduced metabolism associated with poor PF, but these results will have to be validated in a more numerous population, ideally including neuropathology-confirmed cases, or at least a higher number of biomarker-confirmed diagnoses. Our sample’s neurological and neuropsychological profile and FDG-PET pattern were very typical for PSP (and in all patients with a biomarker results ruled out amyloid), but pathology would guarantee the highest degree of diagnostic certainty (Höglinger et al., 2017).

In addition to contributing to knowledge about the source of reduced fluency in PSP, we believe that our findings also have potentially useful implications for the clinical management of the disease. Firstly, we have provided evidence in support of the specificity of Letter fluency as a tool for the assessment of frontal executive dysfunction in this disorder. Secondly, we have better defined the anatomical target for transcranial stimulation techniques, which are starting to show promise for treating fluency deficits in PSP (Madden et al., 2019; Valero-Cabré et al., 2019), a disease that is still completely lacking effective pharmacotherapy.

## Data availability statement

Study data are available from the corresponding author upon reasonable request. Requests to access the datasets should be directed to VI, valeria.isella@unimib.it.

## Ethics statement

The studies involving human participants were reviewed and approved by Comitato Etico Brianza. The patients/participants provided their written informed consent to participate in this study.

## Author contributions

VI conceived and organized the research project, designed and executed the statistical analysis, and wrote the manuscript draft. DL organized and executed the research project, executed the statistical analysis, and wrote the manuscript draft. FF and SM executed the research project and reviewed the draft. CC organized the research project and reviewed the draft. IA and CF conceived and organized the research project and reviewed the draft. All authors contributed to the manuscript and approved the final version of the manuscript.

## Footnotes

1
https://www.fil.ion.ucl.ac.uk/spm
