# Supplementary material for: Reduced phonemic fluency in progressive supranuclear palsy is due to dysfunction of dominant BA6
Source: Front Aging Neurosci. 2022 Sep 8;14:969875. doi: 10.3389/fnagi.2022.969875 (PMC9492952; doi:10.3389/fnagi.2022.969875)
Supplement: Supplementary file 1 [file Table_1.DOCX]

**SUPPLEMENTARY TABLE 1. Peak coordinates, cluster extents and t and p values of clusters of hypometabolism found in patients with PSP compared with healthy controls (p < 0.05 family-wise-error corrected, minimum cluster size: 100 voxels).**

| **Cluster size (n. voxels)** | **p_FWE-corr_, t** | **MNI coordinates** | **Region (Brodmann area)** |
| --- | --- | --- | --- |
| 6030 | 0.000, 6.29 | -18, 10, 46 | Left Superior frontal gyrus |
|  | 0.005, 5.31 | 24, 20, 38 | Right Superior frontal gyrus |
|  | 0.002, 5.62 | -12, 26, 38 | Left Superior frontal gyrus (BA6) |
|  | 0.003, 5.50 | 16, 38, 54 | Right Superior frontal gyrus (BA8) |
|  | 0.025, 4.82 | -2, 2, 34 | Left Mid cingulate (BA24) |
| 1545 | 0.003, 5.49 | 16, -4, 16 | Right Caudate |
|  | 0.049, 4.71 | -18, 0, 10 | Left Putamen |
|  | 0.020, 4.90 | 2, -12, 6 | Right Thalamus |
|  | 0.048, 4.73 | -4, -22, 10 | Left Thalamus |
|  | 0.029, 4.76 | -10, -16, -10 | Left Midbrain |
| 855 | 0.025, 4.82 | -46, 16, -6 | Left Inferior frontal gyrus (BA47) |
|  | 0.048, 4.73 | -58, 16, 18 | Left Inferior frontal gyrus (BA44) |
| 740 | 0.029, 4.76 | 58, 14, 8 | Right Inferior frontal gyrus (BA44) |
| 200 | 0.029, 4.76 | 60, -48, 26 | Right Supramarginal gyrus (BA40) |

**SUPPLEMENTARY TABLE 2. Peak coordinates, cluster extents and t and p values of clusters of hypometabolism associated with poor Letter fluency and copy of Rey-Osterrieth Complex Figure (ROCF) (p < 0.001 uncorrected, minimum cluster size: 100 voxels).**

| **Cluster size (n. voxels)** | **p_0.001uncorr_, t** | **MNI coordinates** | **Region (Brodmann area)** |
| --- | --- | --- | --- |
| **Letter fluency:** |  |  |  |
| 1544 | 0.000, 4.90 | -12, 4, 74 | Left Supplementary motor area (BA6) |
|  | 0.000, 4.66 | -32, 2, 58 | Left Superior/Middle frontal gyrus |
| **Copy of ROCF:** |  |  |  |
| 1910 | 0.000, 4.59 | 16, -72, 62 | Right Superior parietal gyrus |
|  | 0.000, 4.93 | -4, -70, 60 | Left Precuneus |
